# Impact of Trunk Versus Skeletal Muscle Mass Gain on Balance Improvement in Patients with Cerebral Infarction

**DOI:** 10.31662/jmaj.2025-0298

**Published:** 2025-10-03

**Authors:** Keisuke Sato, Naokazu Arasaki, Shota Agena, Seiji Tanaka, Masaki Koike, Takahiro Ogawa

**Affiliations:** 1 LIM projects Inc., Kitanakagusuku, Nakagami District, Okinawa, Japan; 2 Department of Rehabilitation Medicine, Aichi Medical University, Nagakute, Aichi, Japan; 3 Kobe College of Medical Welfare, Sanda, Hyogo, Japan

**Keywords:** stroke, trunk muscle mass, skeletal muscle mass, balance function, rehabilitation

## Abstract

**Introduction:**

Increased muscle mass may positively influence the recovery of balance function. In this study, we aimed to investigate the relationship between changes in muscle mass and improved balance in patients with cerebral infarction.

**Methods:**

This study included patients with cerebral infarction aged ≥65 years. The Berg balance scale (BBS) was used to evaluate balance function upon admission and discharge.

Participants were categorized into two groups based on BBS improvement: those who achieved the minimal clinically important difference (BBS improvement group; 213 participants, 73.4%) and those who did not (no BBS improvement group; 77 participants, 26.6%). Multiple regression analyses were performed, with increase in BBS score as the primary variable of interest and gains in skeletal muscle mass index (SMI) (Model 1), trunk muscle mass index (TMI) (Model 2), and SMI and TMI (Model 3) as explanatory variables.

**Results:**

A total of 290 participants were analyzed. BBS gain exhibited an independent association with TMI gain (coefficient = 3.72, 95% confidence interval [CI] = 0.99-6.45, p = 0.008); however, no significant association was observed with SMI gain (coefficient = 0.03, 95% CI = −2.28 to 2.33, p = 0.983). Furthermore, in Model 3, TMI gain (coefficient = 4.28, 95% CI = 1.35-7.20, p = 0.004) was independently linked to BBS gain. However, in the subgroup analyses stratified by tertiles of rehabilitation volume, this association was not statistically significant in any subgroup.

**Conclusions:**

In patients with cerebral infarction, an increase in TMI was associated with greater improvements in balance function during hospitalization. These results suggest a potential role for trunk muscle mass in supporting balance recovery; however, owing to the observational nature of the study, the findings should be interpreted with caution and viewed as hypothesis-generating. In addition, the lack of association in the subgroup analyses underscores the potential influence of confounding factors, such as the amount and content of rehabilitation, and highlights the need for future studies to control for these variables.

## Introduction

Stroke can affect the central nervous system, presenting significant challenges for patients with stroke in terms of physical functionality. Among the factors influencing functional capacity and quality of life, balance plays a crucial role ^(1)^, with studies indicating that 78% of stroke survivors experience impaired balance ^(2)^. The ability to maintain balance not only influences fundamental tasks, such as standing, walking, and navigating, but also affects activities of daily living (ADLs) ^(3), (4)^. In addition, balance function has been identified as a predictor for length of hospital stay and discharge destination ^(5)^. These findings clearly underscore the substantial impact that balance impairment can have on the lives of patients with stroke, thus emphasizing the pivotal role of balance enhancement in restoring a healthy lifestyle. However, proactive and effective measures to improve balance are essential for fostering physical function recovery and enhancing overall quality of life.

Research indicates that patients with stroke often experience muscle mass loss, which tends to worsen over time during the acute phase of stroke ^(6)^. This decline in muscle mass can precipitate health complications, with studies linking muscle loss and sarcopenia in patients with stroke to reduced ADL ^(7), (8)^. Furthermore, skeletal muscle mass at stroke onset or admission to recovery units has emerged as a key predictor of gait function and walking speed ^(9), (10)^, underscoring the importance of regular muscle mass assessments in stroke management. Moreover, reduced muscle mass is closely linked to decreased physical function, potentially resulting in limitations in ADL. Therefore, accurately monitoring changes in muscle mass and implementing appropriate interventions in rehabilitation programs for patients with stroke is crucial.

Insufficient muscle mass may compromise the essential physical functions necessary for maintaining balance and increase the risk of falls. A study found that lower skeletal muscle mass index (SMI) upon admission to the recovery unit among patients with cerebral infarction is associated with smaller improvements in balance function during hospitalization, with trunk muscle mass index (TMI) demonstrating a stronger correlation than SMI ^(11)^. In addition, TMI has been found to be more strongly associated with functional independence measure (FIM) motor scores at discharge than SMI in patients with acute stroke ^(12)^. Despite these findings, no studies have explored the relationship between improved muscle mass and balance during hospitalization. Therefore, it is imperative to investigate how changes in muscle mass during hospitalization impact balance improvements. This study aimed to elucidate the relationship between muscle mass changes and improvements in balance function among patients with cerebral infarction. By investigating the extent to which changes in muscle mass during a patient’s hospitalization period contribute to improvements in balance function, this research aimed to inform the development of rehabilitation programs and improved patient care.

## Materials and Methods

### Ethics

This study adhered to the principles outlined in the Declaration of Helsinki and was conducted in accordance with the ethical guidelines of Aichi Medical University Hospital. The Ethical Committee of Aichi Medical University Hospital approved the study protocol before the collection and analysis of patient data (approval number 2023-213). Given the retrospective nature of this study, the requirement for informed consent was waived. However, a notice was posted on the hospital’s website, providing participants with the option to opt out of the study. In addition, patients were given the autonomy to withdraw from the study at any point.

### Study design and participants

This retrospective observational study focused on patients with cerebral infarction who were admitted to a convalescent rehabilitation hospital and experienced difficulty walking independently. Inclusion criteria encompassed patients aged ≥ 65 years with cerebral infarction who presented between May 2018 and January 2023. Exclusion criteria comprised patients with severe stroke at admission, those lacking data on SMI and TMI using a bioelectrical impedance analyzer (BIA) at admission and discharge, and those without an assessment of balance function.

### Data collection

Patient characteristics, including age, sex, stroke type, history of stroke, history of fractures, days from onset to hospital admission, body mass index, National Institutes of Health Stroke Scale (NIHSS) score ^(13)^, SMI, TMI, Berg Balance Scale (BBS) score ^(14)^, and FIM score ^(15)^, were extracted from medical records. Stroke severity was assessed using the NIHSS, with scores ranging from 0 to 42 points, where a higher score indicates greater neurologic severity. In addition, the rehabilitation volume per day (min/day) and total rehabilitation volume (min) were computed based on patients’ medical data and assessed using the FIM, with scores ranging from 18 to 126 points. The FIM encompasses 13 physical and five cognitive items rated on a scale of 1 to 7, with higher scores indicating greater independence in ADL.

### Body composition assessment

Bioelectrical impedance analysis was conducted on all participants using a segmental multifrequency BIA (InBody S10; InBody Japan, Tokyo, Japan). Limb skeletal muscle mass and trunk muscle mass were divided by the square of the height to determine SMI and TMI, respectively. Trunk muscle mass comprises skeletal and visceral muscles and remains consistent, except in conditions inappropriate for BIA measurements ^(16)^. Thus, trunk muscle mass measured using BIA under normal body conditions reflects actual muscle mass.

BIA has been widely used in clinical and research settings for the assessment of body composition. In particular, it has demonstrated acceptable diagnostic validity for sarcopenia in Asian populations, as shown in recent studies ^(17)^. Although patients with stroke may experience fluid imbalance because of factors such as edema or malnutrition, BIA has also been applied in many studies targeting this population ^(11), (12), (18), (19)^. In our study, we standardized the measurement conditions as much as clinically feasible to reduce variability associated with hydration status. Specifically, all measurements were performed in the supine position after approximately 15 minutes of rest, at a consistent time of day and room temperature. Patients fasted for at least 2 hours before measurement, which was considered the maximum achievable in a rehabilitation setting. Measurements were conducted using the same equipment and protocols at admission and discharge.

### Balance function assessment

The attending physical therapist used the BBS to evaluate balance function at admission and discharge. The BBS evaluations were performed by unblinded physical therapists who were aware of the patients’ clinical status, which may have introduced observer bias. However, to minimize variability and improve consistency, all assessments were performed according to a standardized protocol based on the official BBS manual, and physical therapists were pre-trained in its implementation. The BBS, a widely used rating scale for assessing balance impairment ^(14)^, comprises 14 items that encompass various aspects of balance, such as maintaining a standing position while moving (items 1, 4, and 10), static standing (item 2), and static sitting posture (item 3). Each item is scored on a 5-level ordinal scale, ranging from 0 (unable to perform or requiring support) to 4 (normal performance), yielding a total score ranging from 0 to 56 points. A higher score indicates a better balance function.

The minimum clinically important difference (MCID) for change in BBS score was reported as four points ^(20)^. Based on this threshold, we defined the “BBS improvement group” as patients who exhibited a BBS gain of ≥ 4 points. Patients were divided into two groups based on BBS gain: the BBS improvement group (BBS gain ≥ 4 points) and the no BBS improvement group (BBS gain < 4 points).

### Primary and secondary outcomes

The primary outcome was the BBS gain, calculated as the difference between BBS scores at discharge and admission. The secondary outcome was an increase in TMI. Patients were stratified into two groups based on their TMI change during hospitalization: those with increased TMI (TMI gain > 0.01) and those without increased TMI (TMI gain: 0 or < 0.01). This threshold was used to distinguish between any measurable increase versus no increase in TMI during hospitalization. To test the robustness of this threshold, we conducted a sensitivity analysis using two additional cut-off values: >0.05 kg/m^2^ and >0.10 kg/m^2^.

### Sample size calculation

The study’s sample size was calculated using the Power and Sample Size Calculation software (version 3.1.2) ^(21)^. Considering an MCID of four points on the BBS ^(20)^, a standard deviation of the population mean of 10.6 points ^(22)^, an α error of 0.05, and a power of 0.8, our analysis indicated that at least 268 participants needed to be enrolled, accounting for a 20% loss to follow-up.

### Statistical analyses

Continuous data, including parametric and nonparametric data, were presented as medians (interquartile ranges) or means (standard deviations) and assessed for normality using the Shapiro-Wilk test. Categorical variables were expressed as counts and percentages. The chi-square and Mann-Whitney U tests were used to compare categorical and continuous data, respectively.

Multiple regression analysis was performed with BBS score gain as the dependent variable and SMI gain (Model 1), TMI gain (Model 2), and SMI gain and TMI gain (Model 3) as explanatory variables. Adjustments were made for age, sex, NIHSS score, initial BBS score, FIM score, length of the hospital stay, and rehabilitation volume, known to be associated with balance function ^(11), (23)^. History of stroke and type of stroke, which showed significant differences in univariate analysis, were also adjusted as explanatory variables. In addition, a subgroup analysis was performed by stratifying participants into tertiles according to daily rehabilitation volume (low, middle, and high). Furthermore, the analysis was repeated, with increased TMI as the dependent variable. All statistical analyses were conducted using R software (version 1.55) ^(24)^, with statistical significance set at 5%.

## Results

**Figure 1.**
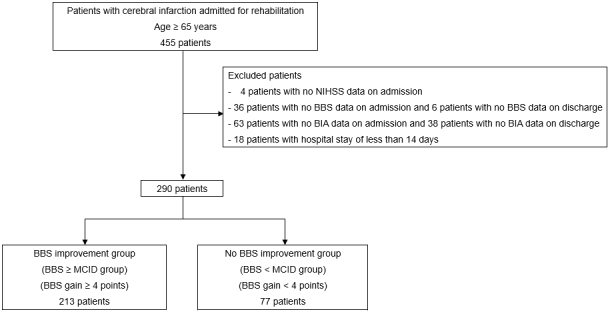
Patient inclusion diagram. BBS: Berg Balance Scale; BIA: bioelectrical impedance analysis; MCID: minimal clinically important difference; NIHSS: National Institutes of Health Stroke Scale.

A total of 455 patients were enrolled during the study period. Among them, 147 patients were excluded because of missing data, and 18 patients had a length of hospital stay of <14 days. Consequently, only 290 participants were included in this study (Figure 1). Table 1 summarizes the baseline characteristics and outcomes of the participants. The mean age of the patients was 78.7 ± 7.9 years, and 52.4% were men. Among the 290 participants, 213 (73.4%) individuals achieved a change of ≥4 points on the BBS and were classified as the BBS improvement group, whereas 77 (26.6%) individuals showed a change of <4 points and were categorized as the no BBS improvement group. There were no significant differences between the two groups in terms of age, sex, BMI at admission, NIHSS score, or the number of days from stroke onset to admission to the rehabilitation hospital (all p > 0.05). However, the FIM score at admission was significantly higher in the BBS improvement group than in the no BBS improvement group (61.1 vs. 54.3, p = 0.013). Regarding discharge outcomes, the daily duration of rehabilitation was significantly longer in the BBS improvement group (141.8 minutes vs. 131.4 minutes, p < 0.001). In addition, the FIM score at discharge (93.4 vs. 76.8, p < 0.001) and the FIM gain (32.3 vs. 22.6, p < 0.001) were significantly higher in the BBS improvement group.

**Table 1.** Baseline Characteristics and Outcomes between Groups with or without Improved BBS.

		BBS improvement	No BBS improvement	p-Value
	Overall	group	group	
	(n = 290)	(BBS ≥ MCID group)	(BBS < MCID group)	
		(n = 213)	(n = 77)	
On admission				
Age (years)	78.7 (7.9)	78.9 (7.9)	78.3 (8.0)	0.592
Sex, n (%)				0.183
Men	152 (52.4)	117 (54.9)	35 (45.5)	
Women	138 (47.6)	96 (45.1)	42 (54.5)	
Type of stroke, n (%)				0.029
Lacunar infarction	91 (31.4)	73 (34.3)	18 (23.4)	
Atherothrombotic cerebral infarction	150 (51.7)	100 (46.9)	50 (64.9)	
Cardiogenic cerebral embolism	49 (16.9)	40 (18.8)	9 (11.7)	
History of stroke, n (%)	90 (31.0)	57 (26.8)	33 (42.9)	0.014
History of fracture, n (%)	38 (13.1)	26 (12.2)	12 (15.6)	0.437
Days from onset to rehabilitation hospital (d)	22.3 (19.7)	21.8 (18.9)	23.6 (22.0)	0.474
BMI (kg/m^2^)	23.5 (3.8)	23.5 (3.6)	23.7 (4.4)	0.752
NIHSS score (points)	5 [2-9]	5 [2-9]	6 [2-14]	0.287
FIM score (points)	59.3 (20.7)	61.1 (19.6)	54.3 (22.8)	0.013
At discharge				
Length of hospital stay (d)	87.4 (38.3)	89.9 (36.4)	80.5 (42.6)	0.063
Rehabilitation volume (min/d)	139.0 (19.8)	141.8 (19.5)	131.4 (18.9)	<0.001
BMI (kg/m^2^)	23.0 (3.5)	23.0 (3.3)	23.2 (4.1)	0.535
FIM score (points)	89.0 (29.7)	93.4 (26.5)	76.8 (34.4)	<0.001
FIM gain (points)	29.7 (15.9)	32.3 (15.1)	22.6 (15.8)	<0.001

Values shown as means (SD) or medians [25th-75th percentiles] or number (%). BBS, Berg balance scale; BMI, body mass index; FIM, functional independence measure; MCID, minimal clinically important difference; NIHSS, National Institutes of Health Stroke Scale; SD: standard deviation.

Table 2 compares SMI, TMI, and BBS between the two groups. The SMI on admission showed no significant difference between the two groups in men (6.51 vs. 6.43 kg/m^2^, p = 0.659); however, in women, the BBS improvement group had significantly higher values than the no BBS improvement group (5.27 vs. 4.90 kg/m^2^, p = 0.021). Regarding the TMI, no significant differences were observed between the groups in either sex (all p > 0.05). No significant difference in SMI at discharge was found between the groups in men (6.64 vs. 6.63 kg/m^2^, p = 0.970), whereas in women, the SMI remained significantly higher in the BBS improvement group (5.40 vs. 5.04 kg/m^2^, p = 0.023). Although there were no significant differences in SMI gain between the groups in either sex (all p > 0.05), a significant difference in TMI gain was observed among women, with the BBS improvement group showing an increasing trend compared with the no BBS improvement group (0.06 vs. −0.15 kg/m^2^, p = 0.013).

**Table 2.** Comparison of SMI, TMI, and BBS between Groups with and without Improved BBS.

	Overall	BBS improvement group	No BBS improvement group	p-Value
On admission				
SMI (kg/m^2^)				
Men	6.49 (0.99)	6.51 (0.96)	6.43 (1.12)	0.659
Women	5.16 (0.85)	5.27 (0.75)	4.90 (1.01)	0.021
TMI (kg/m^2^)				
Men	7.55 (1.40)	7.62 (1.51)	7.35 (0.96)	0.324
Women	6.35 (0.92)	6.35 (0.89)	6.35 (1.01)	0.978
BBS score (points)	24.2 (18.4)	23.8 (16.8)	25.3 (22.2)	0.547
At discharge				
SMI (kg/m^2^)				
Men	6.64 (0.92)	6.64 (0.90)	6.63 (1.01)	0.970
Women	5.29 (0.86)	5.40 (0.71)	5.04 (1.10)	0.023
SMI gain (kg/m^2^)				
Men	0.15 (0.48)	0.14 (0.50)	0.21 (0.43)	0.459
Women	0.14 (0.50)	0.13 (0.47)	0.14 (0.57)	0.968
TMI (kg/m^2^)				
Men	7.52 (1.38)	7.57 (1.48)	7.36 (1.00)	0.434
Women	6.34 (0.90)	6.41 (0.91)	6.19 (0.87)	0.190
TMI gain (kg/m^2^)				
Men	−0.03 (0.36)	−0.04 (0.34)	0.01 (0.40)	0.471
Women	−0.00 (0.47)	0.06 (0.49)	−0.15 (0.38)	0.013
BBS score (points)	35.3 (18.7)	38.6 (16.0)	25.9 (22.2)	<0.001
BBS gain (points)	11.0 (11.2)	14.8 (10.6)	0.6 (4.2)	<0.001
Men	11.9 (10.9)	15.2 (10.4)	0.9 (1.7)	<0.001
Women	10.1 (11.6)	14.4 (10.9)	0.3 (5.5)	<0.001

Values shown as means (SD). BBS: Berg balance scale; SD: standard deviation; SMI: skeletal muscle mass index; TMI: trunk muscle mass index.

Table 3 shows the outcomes of multiple linear regression analysis for BBS score gain. BBS score gain was independently associated with TMI gain (coefficient = 3.72, 95% confidence interval [CI] = 0.99-6.45, p = 0.008) but not with SMI gain (coefficient = 0.03, 95% CI = −2.28 to 2.33, p = 0.983). In Model 3, TMI gain (coefficient = 4.28, 95% CI = 1.35-7.20, p = 0.004) remained independently associated with BBS score gain. To further explore the robustness of the observed association between TMI gain and BBS improvement, we conducted subgroup analyses by stratifying patients into tertiles based on daily rehabilitation volume (Supplementary Tables 1 and 2). Multiple linear regression analyses within each tertile revealed that TMI gain was not significantly associated with BBS gain in any group (all p > 0.05), although the effect size was numerically larger in the high rehabilitation group.

**Table 3.** Multiple Linear Regression Analysis for BBS Gain.

	Model 1			Model 2			Model 3		
	B	95% CI	p-Value	B	95% CI	p-Value	B	95% CI	p-Value
Age	−0.09	−0.24 to 0.06	0.258	−0.11	−0.26 to 0.04	0.153	−0.11	−0.26 to 0.04	0.147
Sex*	2.28	−0.01 to 4.57	0.051	2.27	0.01-4.53	0.049	2.33	0.07-4.59	0.044
Lacunar infarction	−0.69	−4.29 to 2.92	0.708	−0.27	−3.83 to 3.30	0.883	−0.30	−3.87 to 3.27	0.869
Atherothrombotic cerebral infarction	−1.05	−4.28 to 2.19	0.525	−1.22	−4.41 to 1.97	0.452	−1.12	−4.32 to 2.08	0.491
History of stroke	4.08	1.61-6.55	0.001	4.68	2.21-7.16	<0.001	4.72	2.24-7.19	<0.001
NIHSS score on admission	−0.42	−0.68 to −0.16	0.002	−0.40	−0.66 to −0.15	0.002	−0.41	−0.67 to −0.16	0.002
BBS score on admission	−0.40	−0.49 to −0.31	<0.001	−0.41	−0.50 to −0.32	<0.001	−0.41	−0.50 to −0.32	<0.001
FIM score on admission	0.17	0.08-0.27	0.001	0.17	0.08-0.27	<0.001	0.17	0.07-0.27	0.001
Length of hospital stay	0.03	0.00-0.07	0.038	0.03	0.00-0.06	0.044	0.03	0.00-0.06	0.044
Rehabilitation volume	0.11	0.05-0.16	<0.001	0.11	0.05-0.16	<0.001	0.11	0.05-0.16	<0.001
SMI gain	0.03	−2.28 to 2.33	0.983				−1.27	−3.71 to 1.17	0.307
TMI gain				3.72	0.99-6.45	0.008	4.28	1.35-7.20	0.004

Model 1 adjusted for: age, sex, stroke subtype (lacunar infarction, atherothrombotic cerebral infarction), history of stroke, NIHSS score on admission, BBS score on admission, FIM score on admission, length of hospital stay, rehabilitation volume, and SMI gain. Model 2 adjusted for the same variables as Model 1, except that SMI gain was replaced with TMI gain. Model 3 included all variables from Model 1 and Model 2, with SMI gain and TMI gain included. *Men were defined as “1”, and women were defined as “0”. BBS: Berg balance scale; CI: confidence interval; FIM: functional independence measure; NIHSS: National Institutes of Health Stroke Scale; SMI: skeletal muscle mass index; TMI: trunk muscle mass index.

Table 4 presents the outcomes of multivariate logistic regression analyses for increased TMI, using three different thresholds (>0.01, >0.05, and >0.10 kg/m^2^). Across all thresholds, male sex and lower TMI at admission consistently emerged as significant predictors of TMI increase. These findings suggest that the observed associations are robust and not dependent on the specific cutoff used to define TMI gain.

**Table 4.** Multivariate Logistic Regression Results for Increased TMI Using Different Thresholds.

Threshold of TMI gain (>0.01 kg/m^2^)	Odds ratio	95% CI	p-Value
Age	0.996	0.96-1.03	0.799
Sex*	1.850	1.06-3.26	0.032
NIHSS score on admission	0.989	0.94-1.05	0.700
TMI on admission	0.628	0.48-0.82	0.001
FIM score on admission	0.991	0.97-1.01	0.387
Length of hospital stay	0.997	0.99-1.00	0.361
Rehabilitation volume	0.997	0.99-1.01	0.664
Threshold of TMI gain (>0.05 kg/m^2^)			
Age	1.000	0.97-1.04	0.883
Sex*	1.890	1.07-3.36	0.029
NIHSS score on admission	0.994	0.94-1.05	0.825
TMI on admission	0.626	0.48-0.82	0.001
FIM score on admission	0.993	0.97-1.01	0.480
Length of hospital stay	0.999	0.99-1.01	0.835
Rehabilitation volume	1.000	0.99-1.01	0.975
Threshold of TMI gain (>0.10 kg/m^2^)			
Age	1.020	0.98-1.05	0.356
Sex*	1.800	1.00-3.22	0.049
NIHSS score on admission	1.010	0.95-1.07	0.731
TMI on admission	0.687	0.52-0.90	0.007
FIM score on admission	1.000	0.98-1.02	0.988
Length of hospital stay	1.000	1.00-1.01	0.644
Rehabilitation volume	0.999	0.99-1.01	0.823

This multivariate logistic regression model was adjusted for age, sex, NIHSS score on admission, TMI on admission, FIM score on admission, length of hospital stay, and rehabilitation volume. *Men were defined as “1”, and women were defined as “0”. CI: confidence interval; FIM: functional independence measure; NIHSS: National Institutes of Health Stroke Scale; TMI: trunk muscle index.

## Discussion

In this study, we investigated the association between changes in TMI or SMI and balance function among patients with cerebral infarction. Our analysis revealed that TMI gain but not SMI gain was independently associated with improvement in BBS scores. In addition, in men participants, higher baseline TMI was associated with greater in-hospital increases in TMI.

Patients with increased TMI during hospitalization demonstrated greater improvements in balance at discharge. A previous investigation indicated that SMI and TMI at admission were linked to changes in BBS scores among patients with post-cerebral infarction admitted to a convalescent rehabilitation ward ^(11)^. In addition, it suggested that TMI might exert a more substantial influence on BBS improvement than SMI at admission ^(11)^. However, the relationship between increased muscle mass and improved balance during hospitalization was not explored. Our findings revealed that an increase in SMI showed no correlation with improved balance function, whereas an increase in TMI was independently associated with it. Previous studies have underscored the involvement of trunk muscles in postural retention ^(25)^ and highlighted an association between trunk muscle morphology, strength, and the risk of falls ^(26)^. In addition, activation of abdominal muscles has been shown to increase abdominal muscle thickness and improve balance and walking capabilities ^(27)^. Moreover, an increase in trunk muscle thickness has been associated with a broader range of postural stability and reduced postural sway, particularly, in the longitudinal and transverse directions ^(28)^. Furthermore, increased TMI has been linked to improved trunk function ^(18)^. Taken together, these results suggest that increased TMI may support the restoration of trunk function, thereby potentially contributing to balance improvement. However, given the retrospective design of the study, causal relationships cannot be inferred, and the findings should be interpreted with caution. Although trunk function has been linked to ADL ^(29), (30)^, assessing it in early stroke onset can pose significant challenges from the perspective of resting-level issues, such as hemodynamics, comprehension of instructions, and communication difficulties. Thus, our findings may offer insights applicable to many patients with stroke who experienced difficulty in assessing trunk function.

Notably, our analysis found that male sex was significantly associated with an increase in TMI during hospitalization, consistent across all threshold levels in the sensitivity analysis. This observation may reflect general physiological or anatomical differences between sexes, such as higher baseline trunk muscle mass in men or differential hormonal influences; however, these mechanisms were not directly examined in our study. Previous studies have reported that women tend to have lower trunk muscle mass and higher fat content than men ^(31), (32), (33), (34), (35)^ and that intramuscular fat is negatively correlated with muscle strength and thickness ^(36)^. Furthermore, women may be more prone to early muscle loss and have higher rates of sarcopenia post-stroke, whereas men may demonstrate greater gains in muscle mass and strength during rehabilitation ^(37), (38)^. These findings from the literature offer potential explanations, but our results should be interpreted cautiously because our study was not designed to explore underlying sex-specific biological mechanisms. In addition, the present regression analysis (Table 4) revealed that lower TMI at admission was a significant predictor of increased TMI during hospitalization. This finding suggests that patients with lower baseline trunk muscle mass may have had greater capacity to gain muscle mass during the rehabilitation period; however, further studies are needed to verify this interpretation.

This study has several limitations. First, a considerable proportion of eligible patients (147 of 455) were excluded because of missing data, particularly, BIA or BBS measurements. These exclusions may have introduced selection bias, favoring individuals with better physical or cognitive functioning. Consequently, our findings may not be generalizable to the entire population of elderly patients with cerebral infarction undergoing rehabilitation. The included participants may represent a relatively healthier and more cooperative subset of this population, which limits the external validity of the findings. In particular, some patients may have been unable to undergo BBS assessment at admission because of impaired command comprehension associated with severe neurologic deficits, whereas missing BIA data might reflect logistical or operational issues during body composition assessments. Therefore, selection bias should be carefully considered when interpreting our results. Second, the study design did not establish a causal relationship between increased trunk muscle mass and the recovery of balance. Although we adjusted for total rehabilitation time in the main regression models, subgroup analyses stratified by rehabilitation dose revealed that the association between TMI gain and BBS gain was not significant within any group. This implies that unmeasured confounding factors related to rehabilitation intensity or content may have influenced the results. Third, although we adjusted for the total duration of rehabilitation, we were unable to account for several important confounding factors, including the content and quality of rehabilitation, as well as nutritional status, cognitive function, medication use, and muscle strength. Although we acknowledged the potential impact of nutrition and pharmacological interventions, relevant data such as C-reactive protein, albumin, or medication records were unavailable because of the retrospective nature of the study and limited documentation. Future prospective studies with standardized data collection will be needed to better address these confounding variables. These unmeasured variables may have influenced changes in muscle mass and improvements in balance, thereby affecting the observed associations. Moreover, the use of non-blinded assessors may have introduced observer bias, whereas therapists’ perceptions of recovery potential could have affected rehabilitation delivery. These biases are inherent to retrospective designs and should be considered when interpreting the results.

### Conclusion

Our findings revealed a significant association between increased TMI and balance recovery during hospitalization in patients with cerebral infarction. These results suggest a potential role for trunk muscle mass in supporting balance recovery; however, owing to the observational nature of the study, the findings should be interpreted with caution and viewed as hypothesis-generating. Further prospective or interventional studies are needed to confirm these associations and explore underlying mechanisms.

## Article Information

### Acknowledgments

The authors would like to acknowledge all the patients who agreed to participate in this study.

### Author Contributions

Keisuke Sato and Takahiro Ogawa conceived and designed the study. Keisuke Sato performed the data collection and was supported by Naokazu Arasaki, Shota Agena, Seiji Tanaka, Masaki Koike, and Takahiro Ogawa. Keisuke Sato conducted data analyses, with the support of Takahiro Ogawa. The initial drafts of the manuscript were written by Keisuke Sato and Takahiro Ogawa and critically reviewed and edited by all the other authors. All the authors agree with the final version of the manuscript and are accountable for all aspects of the work.

### Conflicts of Interest

None

### IRB Approval Code and Name of the Institution

The Ethics Committee of Aichi Medical University Hospital approved this study (approval No. 2023―213).

## Supplement

Supplementary Material
