# Supplementary material for: Impact of Trunk Versus Skeletal Muscle Mass Gain on Balance Improvement in Patients with Cerebral Infarction
Source: JMA J. 2025 Oct 3;8(4):1368–76. doi: 10.31662/jmaj.2025-0298 (PMC12598138; doi:10.31662/jmaj.2025-0298)
Supplement: Supplementary Material [file 2433-3298-8-4-1368-s001.pdf]

Supplementary Table 1. Descriptive Statistics by Tertiles of Period of Rehabilitation.

	Period of rehabilitation (min/d)	N	TMI gain (kg/m^2)	BBS gain (points)
T1 (low rehabilitation group)	116.1 (13.6)	96	−0.02 (0.45)	8.3 (9.6)
T2 (middle rehabilitation group)	141.8 (4.6)	97	−0.02 (0.32)	12.1 (11.5)
T3 (high rehabilitation group)	159.0 (6.5)	97	0.00 (0.45)	12.7 (12.1)

Values shown as means (SD).

Abbreviations: TMI, trunk muscle mass index; BBS, Berg Balance Scale.

Supplementary Table 2. Multivariable Regression Analysis of TMI Gain on BBS Gain in Each Tertile Group.

	T1 (Only low rehabilitation group)			T2 (Only middle rehabilitation group)			T3 (Only high rehabilitation group)		
	B	95% CI	P-value	B	95% CI	P-value	B	95% CI	P-value
Age	−0.01	−0.27, 0.25	0.943	−0.20	−0.48, 0.08	0.159	−0.20	−0.47, 0.06	0.135
Sex*	0.23	−3.94, 4.39	0.913	2.94	−1.27, 7.15	0.169	1.84	−2.13, 5.80	0.360
Lacunar infarction	−1.73	−7.58, 4.12	0.558	2.63	−3.33, 8.59	0.382	−2.25	−10.07, 5.56	0.568
Atherothrombotic cerebral infarction	−4.69	−10.02, 0.64	0.084	2.15	−3.27, 7.57	0.433	−1.52	−8.51, 5.48	0.667
History of stroke	1.78	−3.00, 6.55	0.462	3.62	−0.90, 8.14	0.115	7.35	2.99, 11.71	0.001
NIHSS score on admission	−0.25	−0.72, 0.23	0.308	−0.25	−0.71, 0.20	0.272	−0.74	−1.17, −0.31	< 0.001
BBS score on admission	−0.33	−0.51, −0.15	0.001	−0.46	−0.62, −0.29	< 0.001	0.18	−0.04, 0.40	0.105
FIM score on admission	0.30	0.05, 0.56	0.021	0.29	0.05, 0.54	0.021	−0.54	−0.71, −0.36	< 0.001
Length of hospital stay	0.02	−0.03, 0.08	0.395	0.03	−0.03, 0.08	0.381	0.03	−0.03, 0.09	0.269
TMI gain	3.52	−0.92, 7.96	0.119	4.98	−1.53, 11.50	0.132	2.25	−2.15, 6.66	0.312

* Men were defined as "1", and women were defined as "0".

T1–3 adjusted for: age, sex, stroke subtype (lacunar infarction, atherothrombotic cerebral infarction), history of stroke, NIHSS score on admission, BBS score on admission, FIM score on admission, length of hospital stay, and TMI gain.

Abbreviations: TMI, trunk muscle mass index; CI, confidence interval; NIHSS, National Institutes of Health Stroke Scale; BBS, Berg Balance Scale; FIM, Functional Independence Measure.
